# Antioxidant activities of chick embryo egg hydrolysates

**DOI:** 10.1002/fsn3.77

**Published:** 2013-12-18

**Authors:** Hao Sun, Ting Ye, Yuntao Wang, Ling Wang, Yijie Chen, Bin Li

**Affiliations:** 1 Key Laboratory of Environment Correlative Dietology, Huazhong Agricultural UniversityWuhan, 430070, China; 2 College of Food Science and Technology, Huazhong Agricultural UniversityWuhan, 430070, China

**Keywords:** Antioxidant activities, chick embryo egg hydrolysates, nutritive value

## Abstract

Chick embryo egg hydrolysates (CEEH) were obtained by enzymatic hydrolysis of chick embryo egg in vitro-simulated gastrointestinal digestion. The antioxidant activities of CEEH were investigated by employing three in vitro assays, including the 2,2′-azinobis-(3-ethylbenzthiazoline-6-sulfonate)/1,1-diphenyl-2-picrylhydrazyl (ABTS/DPPH)/hydroxyl radical-scavenging assays. The radical-scavenging effect of CEEH (1.0 mg/mL) was in a dose-dependent manner, with the highest trolox equivalent antioxidant capacity for ABTS, DPPH, and that of hydroxyl radicals found to be 569, 2097, and 259.6 *μ*mol/L, respectively; whereas the trolox equivalent antioxidant capacity of unhatched egg for ABTS, DPPH, and that of hydroxyl radicals were found to be 199, 993, and 226.5 *μ*mol/L, respectively. CEEH showed stronger scavenging activity than the hydrolysates of unhatched egg against free radicals such as ABTS, DPPH, and hydroxyl radicals. The antioxidant amino acid analysis indicated that the 14-day CEEH possess more antioxidant amino acids than that of the unhatched egg. In addition, essential amino acids analysis showed that the 14-day CEEH have the highest nutritional value. Combined with the results of the amino acid profiles, CEEH were believed to have higher nutritive value in addition to antioxidant activities than the unhatched egg.

## Introduction

In recent years, it has been acknowledged by food scientists and nutritionists that chick embryo eggs are rich in proteins, amino acids, carbohydrates (glucose, fructose, galactose, arabinose, furanose, etc.), lipids (egg yolk lecithin, etc.), fatty acids (linoleic acid, etc.), constants and trace elements (Ca, Cu, Zn, etc.), vitamins (A, B, C, E), and other nutrients (Lidong et al. 2004; Sparks 2006). During egg hatching, the embryos utilize water, minerals and vitamins for metabolism (Barboza et al. 2009). Nutrients within the embryo change with the incubation time (Ar et al. 1987; Firling et al. 1994; Farkas et al. 1996). Through the mice fed experiments, scholars confirmed that CEEH (chick embryo egg hydrolysates) can increase the spleen index, promote proliferation of mouse lymphocytes, enhance hemolysin activity and macrophage phagocytic capacity as well as prolong exhaustive lethal swimming time (Wu et al. 2012).

Eggs have been proved to possess such biological activities as antioxidant ability, antibacterial, and angiotensin-converting enzyme-inhibitory effect (Pellegrini et al. 2004; Liu et al. 2010a,2010b; Chen et al. 2011). Egg white proteins are widely used as functional and nutritional ingredients in food products and their hydrolysates obtained by protease treatment are water soluble and have high nutritional value (Li-Chan et al. 1995). Research has shown that the egg white protein hydrolysate has good antioxidant activity (Chen and Chi 2011). Two kinds of peptides separated from the egg white protein hydrolysate have strong DPPH radical-scavenging activity (Chen et al. 2012).

Enzymatic hydrolysates of other proteins such as whey protein (Dryáková et al. 2010), rapeseed protein (Pan et al. 2009), and soy protein (Liu et al. 2010a,2010b) have been reported to possess antioxidant activities and their biological activities are related to their amino acid composition, as well as the size and configuration of peptides. Regarding the antioxidant activity, the presence of certain hydrophobic amino acids (His, Trp, Tyr, Phe, Met, Leu, Gly, or Pro) and basic amino acids (Arg or Lys) has been reported to enhance the scavenging activities of peptides (Sarmadi and Ismail 2010). Chick embryo eggs have higher protein content, so CEEH also could have antioxidant capacity.

Based on the above rationales, the objective of our investigation was to examine the in vitro antioxidant activities of CEEH by gastrointestinal proteases and to analyze the free amino acid of CEEH. This paper provides theoretical basis for the application of the chick embryo egg in the area of nutrition and health.

## Material and Methods

### Chemicals

Chicken embryo eggs were obtained from Yukou Industry Co., Ltd. (Beijing, China). 1,1-Diphenyl-2-picrylhydrazyl (DPPH) and 2,2′-azinobis-(3-ethylbenzthiazoline-6-sulfonate) (ABTS) were purchased from Sigma-Aldrich (St. Louis, MO). Ascorbic acid and H_2_O_2_ were purchased from Sinopharm Chemical Reagent Co., Ltd. (Shanghai, China). Pepsin and trypsin were purchased from Nanjing Jiancheng Institute of Biotechnology (Nanjing, China). All solvents and reagents were of analytical grade.

### Gastrointestinal in vitro digestion

In vitro digestion was determined using a method described by Miller et al. (1981). Briefly, a uniform slurry of chicken embryo eggs was prepared using a high-speed homogenizer (1000 rpm, 5 min). The homogenate (1 g) was mixed with 10 mL of pepsin solution (1 g of pepsin in 200 mL of 0.1 mol/L HCl) and the mixture was incubated in a shaking water bath at 37°C for 2 h. The pH of the digestate was increased to 7.5 with 0.1 mol/L NaHCO_3_ after gastric digestion. Further intestinal digestion was performed with the addition of 10 mL of trypsin solution (1 g of trypsin in 200 mL of 0.1 mol/L NaHCO_3_) and incubation in a shaking water bath at 37°C for 2 h. The hydrolysate samples were then incubated in the boiling water for 3–10 min to deactivate enzyme activity. The digestate was centrifuged at 10,000 g for 10 min and separated into two fractions: supernatant, as the bioaccessible fraction, and the residue fraction. The supernatant was freeze-dried (Dura-Dry MP freeze-dryer; FTS Systems, Inc., Ridge, NY), pulverized, and stored at −70°C until analysis.

### Antioxidant capacity determined by radical cation (ABTS^+^)

2,2′-Azinobis-(3-ethylbenzthiazoline-6-sulfonate) assay was based on the method of Re et al. (1999) with a slight modification. ABTS radical cation (ABTS^+^) was produced by the reaction between 7 mmol/L ABTS solution and 2.45 mmol/L potassium persulfate and the mixture was allowed to stand in the dark at room temperature for 12–16 h before use. The ABTS^+^ solution was diluted with ethanol to an absorbance of 0.70 ± 0.02 at 734 nm. After addition of 15 *μ*L of different incubation period of CEEH or trolox or different concentrations of vitamin C (0.02–0.1 mg/mL) standard to 2 mL of diluted ABTS^+^ solution, absorbance was measured at 734 nm 6 min later. Results were expressed as trolox equivalent antioxidant capacity.

### Antioxidant capacity determined by DPPH

The ability to scavenge DPPH free radicals was determined based on the method of Brand-Williams et al. (1995) with minor modifications. Briefly, 0.5 mL of different incubation periods of CEEH or trolox or different concentrations of vitamin C (0.1–0.5 mg/mL) was made to a constant volume and added to 2 mL of a 0.2 mmol/L solution of DPPH in methanol. A control sample containing the same volume of solvent in place of the extract was used to measure the maximum DPPH absorbance. After the reaction was allowed to take place in the dark for 30 min, the absorbance at 517 nm was recorded to determine the concentration of remaining DPPH. Results were expressed as trolox equivalent antioxidant capacity.

### Antioxidant capacity determined by hydroxyl radical

The ·OH-scavenging capacity was measured by monitoring the ·OH-induced oxidation of luminol (Costa et al. 2006). The ·OH was generated by a Fenton system (FeSO_4_–SA–H_2_O_2_). Briefly, 1 mL FeSO_4_ (9 mmol/L) and 1 mL H_2_O_2_ (8.8 mmol/L) were added to 1 mL of different incubation periods of CEEH or trolox or different concentrations of vitamin C (0.04–0.12 mg/mL). One milliliter of salicylic acid (9 mmol/L) was added to the mixture 10 min later. A control sample containing the same volume of solvent in place of hydrolysates was used to measure the maximum absorbance. After the mixture was allowed to stand at room temperature for 30 min, the absorbance at 510 nm was recorded to determine the concentration of remaining hydroxyl radical. Results were expressed as trolox equivalent antioxidant capacity.

### Amino acid analysis

The amino acid composition of CEEH was determined using a method described by Kong and Xiong (2006). One milliliter of the supernatants of CEEH of 0, 6, 11, 14, and 18 days was mixed with 0.25 mL of 2% phenol solution and 0.5 mL of performic acid. After incubation at 20°C for 4 h, 0.2 mL of 16.8% sodium pyrosulfite was added. The samples were subsequently digested in a sealed glass tube with 9 mL of 6.7 N HCl at 110°C for 24 h. The whole digests were transferred into 50 mL volumetric flasks, mixed with 9 mL of 6 mol/L NaOH, and then brought to a volume of 50 mL with 0.02 N HCl. The sample solutions were filtered and loaded on a Model S433D amino acid analyzer (Sykam Corp., Eresing, Germany) for amino acid analysis. Post column reaction with ninhydrin yielded amino acid derivatives, the absorbances of which were measured at 570 and 440 nm. The concentrations of the specific amino acids were determined from their respective absorption intensities, which were calibrated to the known concentrations of amino acid standards.

### Statistical analysis

All tests were done in triplicate, and data were averaged. Standard deviation was also calculated. The data obtained were subjected to multifactor analysis of variance (ANOVA) using SPSS 12.0 (SPSS: Shanghai, China), followed by Duncan's multiple range test to determine the significant difference between samples at the *P* < 0.05 level.

## Results and Discussion

### Antioxidant activity determined by the ABTS method

**Figure 1:**
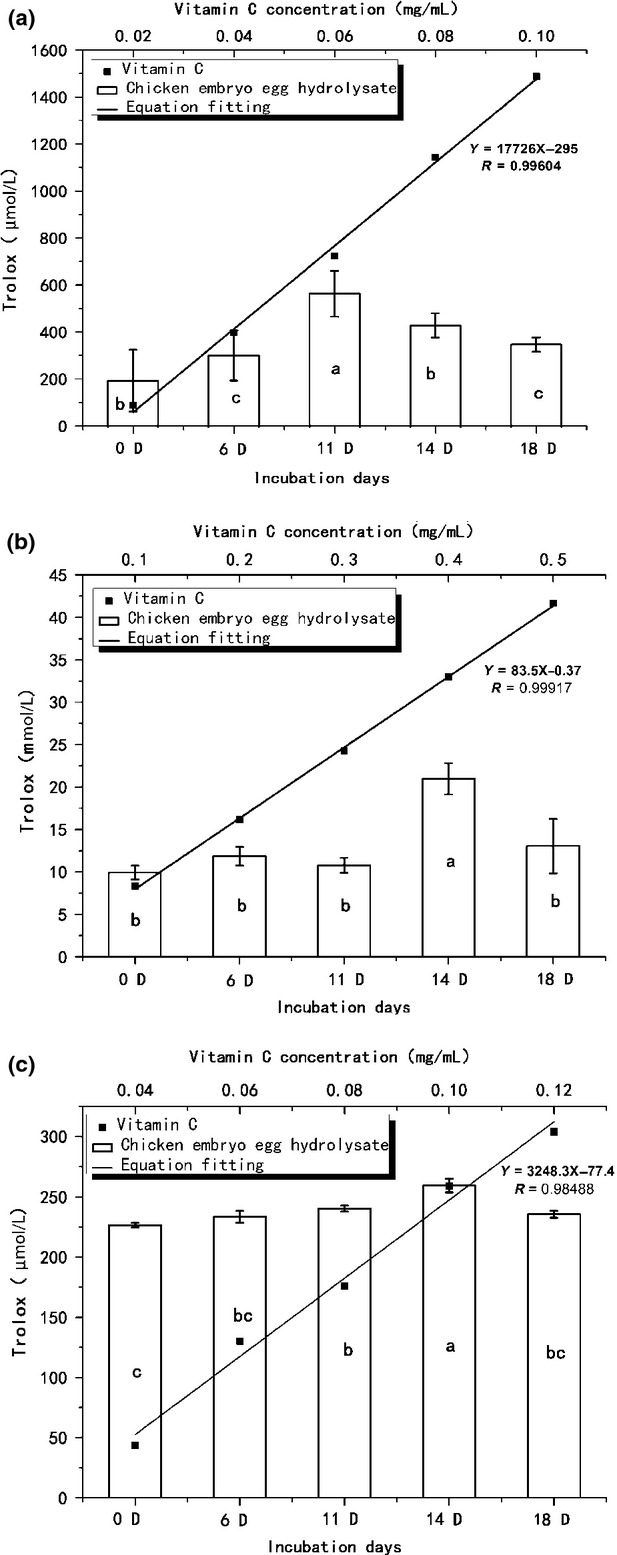
Radical-scavenging activity of different incubation period of CEEH determined by ABTS (a), DPPH (b), and hydroxyl radical (c). The meaning of the equation is a linear fit of vitamin C. Values are means of three replicates ± SD. a–c mean significant difference at *P* < 0.05 by Duncan's test. CEEH, chick embryo egg hydrolysates; ABTS, 2,2′-azinobis-(3-ethylbenzthiazoline-6-sulfonate).

The antioxidant capacity of CEEH was evaluated with the ABTS tests. The free radical-scavenging activity determined by ABTS varied from 199 ± 130 to 569 ± 98 *μ*mol/L trolox (Fig. 1a). Similar results have been reported for peptides isolated from algae protein hydrolysate, the ABTS radical scavenging of which reached 50% at 9.8 ± 0.5 *μ*mol/L. (Sheih et al. 2009).

The ABTS radical-scavenging ability of 11-day CEEH was equal to that of 569.49 *μ*mol/L Trolox, which has the same ABTS radical-scavenging ability with 0.0488 mg/mL of vitamin C. The antioxidant capacity of the 11-day CEEH was stronger than that of the unhatched egg. The result showed that in the early hatching process, the content of substances with ABTS radical-scavenging activity was continuously growing before a decrease was found. The fall in the ABTS radical-scavenging activity was because the free-radical scavenging kind of amino acids and the peptides were absorbed by the chicken embryo.

### Antioxidant activity determined by the DPPH method

DPPH radical has a single electron and shows maximum absorbance at 517 nm. When DPPH radicals encounter a proton-donating substance such as an antioxidant, the radicals would be scavenged and the absorbance is reduced (Jao and Ko 2002). This radical-scavenging assay is quick, convenient, and reproducible, and thus widely used to test the antioxidant activity of some natural compounds (Yamamoto and Kajimoto 1980).

The antioxidant capacity of CEEH was evaluated with the DPPH tests. The free radical-scavenging activity determined by DPPH varied from 9.93 ± 0.81 to 20.97 ± 1.84 mmol/L trolox (Fig. 1b). Similar results have been reported for peptides isolated from rapeseed protein hydrolysates, the DPPH radical-scavenging ability of which reached 75.22% at 1.2 mg/mL (Pan et al. 2009). Liu et al. (2010a,2010b) reported that the peptide fraction possessed DPPH radical-scavenging activity, with the median effective dose (ED_50_, meaning the concentration that scavenges 50% of the initial DPPH radical) value for soy protein hydrolysate being 4.63 mg/mL.

In the DPPH methods, the 14-day CEEH had the same antioxidant capacity with 0.255 mg/mL of vitamin C. The antioxidant capacity of the 14-day CEEH was stronger than that of the unhatched egg. These results revealed that during egg hatching, more DPPH radical-scavenging substances had been produced. The result showed that in the early hatching process, the content of substances with DPPH radical-scavenging activity was continuously growing before a decrease was found. The fall in the DPPH radical-scavenging activity was because the radical-scavenging kind of amino acids and the peptides were absorbed by the chicken embryo.

### Antioxidant activity determined by the hydroxyl radical method

Among oxygen radicals, specifically, the hydroxyl radical is the most reactive and severely damages adjacent biomolecules such as all proteins, DNA, polyunsaturated fatty acid, nucleic acid, and almost any biological molecule it touches. This damage causes aging, cancer and several diseases (Aruoma 1998), and hence, its removal is probably one of the most effective defenses of a living body against various diseases (Lee et al. 2004).

The antioxidant capacity of CEEH was evaluated using the hydroxyl radical-scavenging tests. The free radical-scavenging activity determined by hydroxyl radical varied from 226.5 ± 1.9 to 259.5 ± 5.7 *μ*mol/L trolox (Fig. 1c).

In terms of the scavenging ability of the hydroxyl radical, the 14-day CEEH had the same antioxidant capacity with 0.1 mg/mL of vitamin C. The antioxidant capacity of the 14-day CEEH was stronger than that of the unhatched egg. These results reveal that during egg hatching, more hydroxyl radical-scavenging substances had been produced.

### Amino acid composition

The amino acid composition of the CEEH is shown in Table 1. CEEH had a relatively high content of hydrophobic amino acid such as Phe and Leu. Studies suggested that increased solubility of hydrophobic amino acids in lipids can lead to a better free radical-scavenging capacity (Saiga et al. 2003; Rajapakse et al. 2005; Je et al. 2007). So the different hydrophobic amino acid contents in the four incubation periods in CEEH may cause them to have different free radical-scavenging capacities.

**Table 1:** Free amino acid analysis of the samples.

Free amino acid content (mg/100 mL) (mean ± SD)					
Amino acid	0 Days	6 Days	11 Days	14 Days	18 Days
Asp	1.24 ± 0.13	1.32 ± 0.17	1.34 ± 0.15	1.22 ± 0.11	1.01 ± 0.09
Thr	2.14 ± 0.17	2.5 ± 0.20	2.64 ± 0.24	2.66 ± 0.18	–2
Ser	0.92 ± 0.08	0.9 ± 0.08	1 ± 0.04	0.94 ± 0.03	1.58 ± 0.11
Glu	2.64 ± 0.21	2.92 ± 0.24	2.99 ± 0.19	3.22 ± 0.22	4.62 ± 0.27
Gly	0.54 ± 0.01	0.58 ± 0.02	0.57 ± 0.02	0.73 ± 0.04	0.8 ± 0.01
Ala	1.14 ± 0.14	1.4 ± 0.09	1.46 ± 0.12	1.36 ± 0.12	2.07 ± 0.15
Cys-s	0.39 ± 0.01	0.31 ± 0.02	0.3 ± 0.01	0.38 ± 0.01	0.32 ± 0.02
Val	1.58 ± 0.08	1.8 ± 0.11	1.9 ± 0.1	1.81 ± 0.09	2.88 ± 0.13
Met	2.4 ± 0.15	2.6 ± 0.16	2.7 ± 0.14	2.71 ± 0.19	3.34 ± 0.23
Ile	0.52 ± 0.02	0.58 ± 0.01	0.64 ± 0.04	0.56 ± 0.03	0.78 ± 0.02
Leu	7.18 ± 0.47	8.2 ± 0.52	8.39 ± 0.49	8.62 ± 0.53	9.32 ± 0.61
Tyr	8.42 ± 0.52	9.4 ± 0.5	9.48 ± 0.56	9.5 ± 0.64	9.3 ± 0.67
Phe	15.66 ± 0.54	18.1 ± 0.61	17.96 ± 0.47	18.3 ± 0.68	18.6 ± 0.55
Orn	1.11 ± 0.12	1.21 ± 0.11	1.27 ± 0.04	1.3 ± 0.09	2.1 ± 0.05
Lys	6.42 ± 0.41	7.16 ± 0.38	7.38 ± 0.55	7.9 ± 0.47	7.06 ± 0.22
Pro	0.18 ± 0.01	0.28 ± 0.01	0.18 ± 0.01	0.26 ± 0.01	0.29 ± 0.02
His	0.52 ± 0.04	0.63 ± 0.05	0.64 ± 0.07	0.67 ± 0.02	0.57 ± 0.04
Trp	3.23 ± 0.14	3.22 ± 0.19	3.1 ± 0.08	3.24 ± 0.17	3.18 ± 0.29
Arg	15.06 ± 0.69	17.29 ± 0.78	16.35 ± 0.66	17.44 ± 0.71	12.64 ± 0.54
Total	72.1 ± 3.97	81.2 ± 4.28	81.2 ± 4.05	83.9 ± 4.46	82.5 ± 4.17

SD, standard deviation from duplicate determinations; Asp, aspartic acid; Thr, threonine; Ser, serine; Glu, glutamic acid; Gly, glycine; Ala, alanine; Cys-s, cysteine; Val, valine; Met, methionine; Ile, isoleucine; Leu, leucine; Tyr, tyrosine; Phe, phenylalanine; Orn, ornithine; Lys, lysine; Pro, proline; His, histidine; Trp, tryptophan; Arg, arginine.

1 Incubation period for 18 days of chicken embryo egg hydrolysate no detection of threonine.

**Figure 2:**
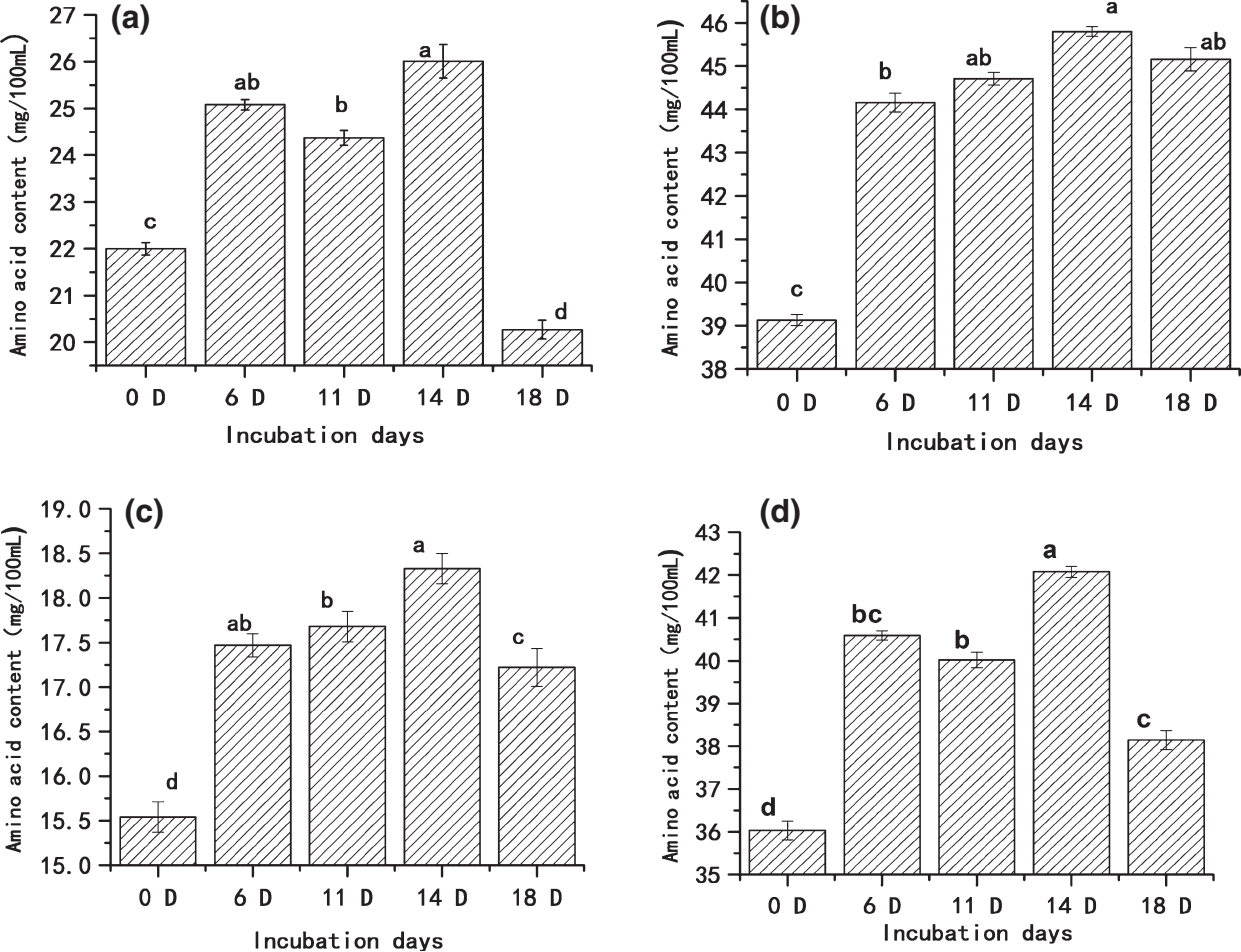
Free amino acid content: (a) basic amino acid (Including Arg, Lys, His). I; (b) the human body essential amino acids (including Lys, Try, Phe, Met, Thr, Ile, Leu, Val). II; (c) radical-scavenging kind of amino acid (including Glu, Cys, Met, Tyr, Lys, Pro, His, Arg). III; (d) antioxidant kind of amino acid (including Tyr, Lys, Pro, His). IV. Values are means of three replicates ± SD. (a–c) mean significant difference at *P *< 0.05 by Duncan's test. Lys, lysine; Tyr, tyrosine; Phe, phenylalanine; Met, methionine; Thr, threonine; Ile, isoleucine; Leu, leucine; Val, valine; Glu, glutamic acid; Cys-s, cysteine; Pro, proline; His, histidine; Arg, arginine.

Studies have shown that some amino acids such as Cys, Ala, and Phe could affect the riboflavin-sensitized photooxidation of ascorbic acid, which could scavenge a certain amount of free radicals (Jung et al. 1996). Other researches had pointed out that in the peptide sequence, His and Pro played important roles in the antioxidative activity and, among the peptides tested, Pro-His-His was the most antioxidative (Chen et al. 1996). As shown in Figure 2, the contents of different types of amino acids in CEEH were studied including those of basic amino acids, essential amino acids, the radical-scavenging kind of amino acid (Jung et al. 1996), and the antioxidant kind of amino acid (Chen et al. 1996; Cao et al. 2009). Compared with the unhatched egg, a significant growth can be found in the content of all the four types (basic amino acid, the human body essential amino acids, radical-scavenging kind of amino acid, and antioxidant kind of amino acid) of amino acids (Fig. 2).

The essential amino acids, including Iso, leucine (Leu), lysine (Lys), methionine (Met), phenylalanine (Phe), threonine (Thr), tyrosine (Try), valine (Val), and histidine (His) must be taken every day because they cannot be synthesized in the human body (Oser 1951; Rodondi et al. 2009). Comparing the essential amino acid level between the sample and the standard protein score is a recognized method used to determine the nutritional value of a raw material (Cao et al. 2009). The content of essential amino acid is higher in CEEH, which means CEEH has good nutritional value. The radical-scavenging kind of amino acid occupies a relatively higher proportion of the total free amino acids, which leads to stronger antioxidant activity of CEEH (Joshi et al. 2001).

Some studies have shown that chick embryo began to form the kidney and bowel function on the 12th day of hatching. Chick embryo began to devour the protein in eggs and protein content reduction is from 60% of the egg weight to 19% (Nir et al. 1993). More amino acids were synthesized in 12-day chick embryo because of the absorption and re-processing of protein in the eggs during the 12th incubation day. With the extension of the incubation period, the content of four types of amino acids increased at first and then decreased. The highest values of four types of amino acids were all exhibited in the 14-day CEEH as shown in Figure 2. It can be seen from the significance analysis that the four types of amino acids of the 14-day CEEH content show a significant difference with the other incubation period. So the chick embryo egg has better nutritional value and antioxidant activity than the unhatched eggs in medium-term hatching.

## Conclusions

The antioxidant activity of CEEH was explored by three methods in this study. The free radical-scavenging activities of CEEH were concentration-dependent. CEEH also contains a high proportion of essential amino acids. CEEH also has a stronger antioxidant property along with better nutritive value than the unhatched egg. In addition, the 11-day and 14-day CEEH had stronger antioxidant activity, which means the incubation medium-term chick embryo eggs have a higher nutritional value. The information included in this study could give useful insight into the potential application of chick embryo eggs in the area of functional food industry. To further illustrate the antioxidant activity of CEEH, further detailed studies on cellular antioxidant experiments are needed.
